# Beyond discrete social decisions: toward continuous dyadic neural dynamics in cooperation and competition

**DOI:** 10.3389/fnsys.2026.1809977

**Published:** 2026-07-31

**Authors:** Qiang Li, Jianmei He, Ziyu Li, Chang Rao, Jing Luo, Junhua Wu

**Affiliations:** 1 College of Education Science, Guizhou Education University, Wudang, Guiyang, China; 2 Guizhou Key Laboratory of Artificial Intelligence and Brain-inspired Computing, Guizhou Education University, Wudang, Guiyang, China; 3 Department of Psychology, Division of Life Sciences and Medicine, University of Science and Technology of China, Hefei, China

**Keywords:** behavior, competition, continuous dynamics, cooperation, social decision-making

## Introduction

In recent research paper published in Communications Psychology, Lewen et al. (2025) introduced a novel and methodologically groundbreaking experimental paradigm i.e., the continuous Cooperation-Competition Foraging (CCF) Game. This paradigm was designed to capture how cooperation and competition emerge dynamically in continuous time and space during real social interactions. By overcoming the limitations of traditional discrete game tasks, the study embedded social decision-making within a naturalistic context by incorporating real-time perception-action coupling, face-to-face visual access, continuous movement, and immediate feedback. This approach offered a more ecologically valid representation of social behavior, aligning it more closely with real-world dynamics of social interactions.

The most significant contribution of this work lies in its shift from “turn-based, discrete, symbolic” frameworks of classical game paradigms to a “continuous, embodied, interactive” perspective grounded in dynamical systems theory, a well-established framework for understanding how complex interacting processes evolve over time in cognitive and social behavior (Gordon et al., 2021; Priorelli et al., 2025; Schöner, 2023; Spivey, 2023; Yoo et al., 2021). In traditional paradigms such as the Prisoner's Dilemma, Ultimatum Game, or Public Goods Game, participants typically make binary or limited-choice decisions in discrete trials where decision-making and action are temporally separated, and there is a lack of real-time visible behavioral cues (Schilbach et al., 2013). Although such setups are conducive to modeling, they are limited in ecological validity and fail to capture continuous flow of information, bodily coordination, and real-time strategic adjustments inherent in real-world social interactions. In contrast, the CCF paradigm developed by Lewen et al. (2025) enabled participants to express their strategies through continuous movement trajectories and adjust their behavior on a millisecond-scale based on the actions of the partner they were interacting with, thus, embodying the concept of “decide-while-acting.” This design more authentically replicates social decision-making processes observed in everyday life, offering a dynamic perspective rather than a static choice model.

Notably, Lewen et al. (2025) demonstrated that dyads spontaneously converge toward relatively stable set-points along a cooperation–competition spectrum, forming cooperative, intermediate, and competitive groups. Rather than adopting extreme strategies, most dyads stabilized around intermediate patterns. Through computational modeling, the authors showed that weighted path minimization, combined with across-cycle predictors such as invitations and target history, predicted dyadic choices with high accuracy (87%). These findings suggest that social coordination emerges from a dynamic interplay between low-level sensorimotor coupling and higher-order strategic preferences.

Taken together, Lewen et al. (2025) contribute not only a sophisticated and thoughtfully designed behavioral paradigm, but also a conceptually important framework for social neuroscience. By integrating real-time visual access, continuous behavioral exchange, immediate feedback, and ecologically relevant information such as action history, current position, and the partner's ongoing behavioral state, the CCF paradigm captures social decision-making in a way that is far closer to natural interactive contexts than traditional laboratory tasks. At the neuroscientific level, the paradigm is equally important because it renders social decision-making tractable not merely as a final choice outcome, but as a temporally extended process composed of transient interaction events and ongoing decision dynamics that can be related to neural activity. In this sense, the CCF paradigm offers a strong foundation for future work aimed at linking continuous social interaction with neural processes across multiple levels of analysis.

## An ingenious and highly informative experimental design

A major strength of this study lies in its remarkably inventive experimental design, which is both technically ambitious and methodologically illuminating. At the center of the paradigm is a large transparent bidirectional display positioned between two participants seated face-to-face, such that the game unfolds on the screen while each participant can simultaneously see the other through it. This arrangement is far more demanding and resource-intensive than conventional computer-based social decision-making paradigms, but precisely for that reason it represents a remarkable methodological commitment to ecological validity. Rather than reducing social choice to isolated responses on separate screens, the design preserves a critical feature of real-world interaction: people make decisions while continuously monitoring one another's facial expressions, body posture, movement tendencies, and moment-to-moment behavioral adjustments. As a result, participants can modify their own choices online in light of the partner's visible reactions, making the unfolding decision process much closer to how cooperation and competition operate in everyday social life. In this sense, the transparent-screen setup is not merely an unusual or clever piece of apparatus. It is a genuinely original and highly informative design innovation that transforms the experimental situation itself, allowing social decisions to emerge under conditions of continuous mutual visibility, embodied responsiveness, and ongoing interpersonal exchange. Precisely because such transparent interactive displays remain rare in behavioral research, their use here is especially creative and consequential, opening a methodological space that most traditional paradigms simply cannot access.

Beyond the ingenuity of the apparatus itself, a second and equally important advantage is that the interaction remains continuously structured and behaviorally informative throughout the task. The game unfolds in a shared two-dimensional space in which both agents, all targets, and the evolving payoff structure are visible to both participants at all times. Crucially, the task preserves continuity from one moment to the next: after a target is collected, new targets appear without resetting the positions of the agents or the remaining items, so the shared spatial situation continues to evolve rather than restarting from scratch. This absence of clearly demarcated trial boundaries is methodologically important, because it allows interaction history to accumulate and shape subsequent decisions. Participants do not repeatedly return to a neutral starting point; instead, each new movement and choice is made against the background of prior coordination, hesitation, concession, or defection. In this way, strategy formation becomes path-dependent, making it possible to observe how cooperative tendencies are built up, stabilized, disrupted, or repaired over time. Under these conditions, each action unfolds through continuous mutual responsiveness: participants can monitor one another's movements, adjust to changing spatial relations, and coordinate their behavior moment by moment rather than making isolated choices in discretized rounds. This makes the task closer to a naturalistic “decide-while-acting” situation, in which action and social evaluation unfold together in real time. Just as importantly, this continuous mutual visibility generates process-level behavioral evidence that conventional round-based paradigms cannot provide. Because movements were tracked at high temporal resolution, the study could analyze invitations, leader-follower dynamics, miscoordination, curved trajectories, and other within-trial interaction patterns, thereby opening access to the microdynamics of social decision-making rather than only its final outcomes.

The target and payoff structure is another particularly insightful feature of the design. At every moment, participants faced one single target worth 7 cents to one player alone and two joint targets that required cooperation but distributed payoffs asymmetrically as 5/2 and 2/5. This is an elegant solution because the total joint payoff of the cooperative and non-cooperative options is held constant, avoiding a trivial incentive bias toward either target type, while the asymmetry within the cooperative options creates the conditions under which one participant can accept a less favorable immediate outcome for the partner's benefit. In turn, this makes prosocial intent behaviorally legible and allows the study to capture whether such short-term concessions are later reciprocated, thereby revealing how reciprocal cooperation can be established and maintained over repeated interactions.

Equally important, the design preserves a genuinely graded strategic space rather than reducing social choice to a forced opposition between cooperation and competition. Because the task repeatedly confronts participants with alternatives that differ in coordination demands and payoff distribution, it allows stable cooperation, partial cooperation, opportunistic defection, and dynamically shifting strategies to emerge within the same framework. This is methodologically important because it prevents behavior from being artificially compressed into a binary classification and instead makes it possible to study how social preferences vary in degree, consistency, and temporal stability. In this way, the experiment is informative not only about whether cooperation occurs, but about how cooperative tendencies are sustained, weakened, or strategically revised over time.

For future behavioral research, the CCF paradigm offers an excellent template for studying social decision-making. Its high temporal resolution, continuous interaction without repeated trial resets, face-to-face visibility, and carefully balanced incentive structure enable researchers to track the real-time unfolding of social decision-making while linking these dynamics to observable outcomes within a single framework, including choices, movement trajectories, coordination signals, fine-grained interaction behaviors such as position holding, waiting, hesitation, and reciprocal adjustment, as well as latent social motives. For that reason, the present design should be regarded not merely as a clever task for one specific study, but as a methodological model for how social decision-making experiments can move beyond static, trial-based paradigms toward richer and more ecologically grounded forms of interaction.

## Promise for social neuroscience: from event-locked neural signatures to continuous decision dynamics

A central contribution of the CCF paradigm introduced in the target article (Lewen et al., 2025) is that it makes social decision-making more tractable for neuroscience by transforming it from a discrete outcome into a temporally extended and behaviorally annotated process. By enabling continuous, face-to-face interaction while generating high-temporal-resolution behavioral measures and model-based estimates of target choice, choice certainty, and spatiotemporal coordination, the paradigm creates quantifiable variables that can be aligned with neural activity. The study therefore does more than demonstrate that cooperation and competition can be examined in a more naturalistic format; it provides a behavioral and computational framework rich enough to support future neural analyses of naturalistic social interaction.

First, this design is especially promising for event-locked social neuroscience, because it reveals interaction events that are largely inaccessible in conventional discrete, trial-based paradigms. In standard economic games, neural activity is typically aligned to coarse markers such as stimulus onset, button press, or feedback (Batten et al., 2024; Guo et al., 2022; Numano et al., 2026; Wang et al., 2021). By contrast, the present paradigm identifies fine-grained social events within continuous interaction, including invitations to cooperate, failed invitations, leader-follower episodes, concurrent approaches to the same target, movement-based hesitation or redirection, and overt breakdowns of coordination in which the two agents commit to different targets. This feature opens the door to asking more specific neural questions: which brain signals accompany a costly cooperative overture, the acceptance or rejection of an invitation, a competitive race for a single target, or a sudden departure from an emerging cooperative convention? In that sense, the paradigm could allow neuroscience to move beyond broad contrasts such as “cooperation vs. competition” toward the neural signatures of socially meaningful micro-events that constitute those broader states.

Second, and perhaps more importantly, the paradigm is well positioned to support the study of decision formation as a continuous neural process. The choices in this paradigm are not isolated selections but unfold through ongoing perception-action loops, mutual adjustment, and “decide-while-acting” dynamics that are shaped not only by the current state of play but also by the recent history of coordination, failed invitations, and reciprocal adaptation. The authors show that model-derived choice uncertainty is systematically related to subsequent trajectory structure: higher uncertainty is associated with more curved trajectories, miscoordination, and target divergence, whereas invitations reduce uncertainty and promote efficient coordination. This is precisely the kind of structure that neuroscience has long needed in order to study how cooperation or competition emerges over time rather than merely how it is reported at the end of a trial. Future work combining this paradigm with neural recording could therefore examine how brain activity evolves as dyads transition from uncertainty to coordination, from mutual monitoring to shared commitment, or from local competition to restored cooperation. As the authors suggest, richer modeling of continuous trajectories may help identify putative decision points that could be used as alignment markers for neural analysis.

Another important advantage is that the paradigm may help dissociate components of social choice that are often collapsed in discrete paradigms. Behavior in this task is shaped not only by overt cooperative preference, but also by movement speed, trajectory efficiency, strategic placement, and within-dyad asymmetries in skill. These factors are especially important because they are characteristic of continuous action spaces yet are not readily captured in conventional discrete tasks. For social neuroscience, this matters because it creates a setting in which social motivation, sensorimotor capacity, and reward optimization can be analyzed jointly without being reduced to a single end-of-trial choice. Neural data collected in such a paradigm could therefore help clarify not simply whether the brain encodes cooperation or competition, but how it negotiates among effort, motor ability, strategic opportunity, and social preference in real time.

From a neuroscientific perspective, the paradigm should be seen not only as an innovative behavioral task, but also as a strong platform for future research. Its combination of continuous interaction, high temporal resolution, explicit behavioral event structure, and formally tractable uncertainty makes it especially well suited for relating neural activity to both moment-to-moment social events and extended decision dynamics. The broader significance of the present work is therefore twofold: it makes an important contribution to behavioral research on social decision-making, while also establishing a paradigm foundation for future investigations of the dynamic processes underlying social choice, including their neural implementation.

## How the CCF paradigm could be integrated with social neuroscience

Integrating the CCF paradigm with social neuroscience requires asking not simply whether it can be studied neurally, but which aspects of the task different neural methods are best suited to capture. Because the paradigm combines rapid event-related transitions, continuous interpersonal adjustment, and slower strategic structure unfolding across repeated collection cycles, it offers a useful bridge between multiple levels of neural analysis. The value of this paradigm, therefore, lies in providing a common behavioral framework within which different components of social decision-making can be linked to different neural signals.

First, the CCF paradigm appears especially well suited for neural methods with high temporal resolution, because the central processes of interest unfold rapidly within and across collection cycles. Rather than being defined primarily by generic task events, the paradigm is organized around fast-changing target choices, invitation-based sensorimotor communication, online trajectory adjustments, and shifts between coordinated and uncoordinated interaction states. Because the authors quantify target-choice uncertainty and classify spatiotemporal trajectories into behaviorally meaningful categories, neural activity can be aligned not only to overt task structure but also to evolving decisional and coordination variables. In this respect, electroencephalography (EEG), magnetoencephalography (MEG), and—where clinically available—electrocorticography (ECoG) are particularly attractive for tracking how participants use the partner's trajectory and positioning in the shared action space to commit, revise, coordinate, or compete in real time.

Functional near-infrared spectroscopy (fNIRS) should be positioned somewhat differently. Relative to EEG, MEG, and ECoG, fNIRS is not optimal for resolving millisecond-scale neural events, because it indexes slower hemodynamic changes rather than direct electrophysiological activity. Its value in this paradigm lies instead in its ability to track superficial cortical dynamics at a coarser but still informative spatiotemporal scale (Yücel et al., 2017), making it suitable for processes that evolve over seconds within and across collection cycles, such as stable cooperative-competitive set points, across-cycle history effects, invitation-based coordination, and the maintenance of established cooperation. Framed in this way, fNIRS is better understood as a complementary method for studying slower, sustained cortical hemodynamics during ongoing interpersonal action, while preserving some spatial differentiation across cortical sites accessible from the scalp.

Second, the CCF paradigm could provide a useful platform for hyperscanning, an approach that is gaining momentum in social neuroscience. Because social interaction is inherently bidirectional and dynamically reciprocal, hyperscanning is particularly well suited to this kind of paradigm, as it enables the simultaneous recording of neural activity from both interacting partners and permits the investigation of neural processes not only within individuals but also between them. In particular, it allows researchers to relate neural activity within and across brains to fine-grained dyadic processes as they unfold in real time, rather than inferring social processes from isolated individuals or coarse task epochs alone. In the present paradigm, the interaction can be decomposed into behaviorally interpretable episodes of social exchange, including the initiation and uptake of invitations, temporary leader-follower organization, coordinated low-uncertainty approaches, and episodes of miscoordination or competition. This event-level structure makes it possible to align within- and between-brain dynamics with specific coordination states, rather than treating the interaction as a single undifferentiated task. Future hyperscanning studies could therefore ask whether inter-brain coupling strengthens when partners converge on a shared decision and weakens when invitations fail, coordination breaks down, or competing trajectories emerge.

Third, fMRI should also be considered a promising extension, even though its temporal resolution is less naturally suited to the task's rapid event structure. Its main value would likely lie not in resolving moment-to-moment online adjustments, but in using its comparatively high spatial resolution to identify the distributed neural systems that support slower and more integrated aspects of performance, including stable strategic tendencies, the valuation of cooperation relative to individual payoff, and the representation of latent social variables that guide joint behavior over time. This seems particularly relevant here, because Lewen et al. (2025) show that dyads converge onto relatively stable strategy regimes, that target choices are highly predictable from a full behavioral model, and that many participants incur a measurable cost of cooperation rather than adopting individually optimal competitive strategies. These findings suggest that the paradigm captures not only fast interpersonal coordination, but also more enduring social-computational structure, making it well suited for fMRI investigations of how such latent variables are represented in the brain. This adaptation would, however, come at the cost of reducing some features of the original behavioral setting, especially direct face-to-face interaction, bodily co-presence, and the full naturalness of reciprocal adjustment. Still, an fMRI implementation that incorporates shared displays, live video feeds of each partner's facial expressions, and a synchronized dual-participant design could preserve at least some of the task's partner-dependent structure, thereby allowing the approach to remain informative. In that case, the contribution of fMRI would lie less in reproducing the paradigm's full ecological richness than in identifying the brain systems underlying the more stable social-computational regularities revealed by the task.

One promising future direction may be multimodal integration. Because the CCF paradigm includes both fast transient events and slower emergent strategy variables, it may be well suited to combining methods that operate across different timescales. EEG-fMRI, for example, could potentially capitalize on the relatively high temporal resolution of EEG and the relatively high spatial resolution of fMRI (Chang and Chen, 2021; Mantini et al., 2010) to relate rapid event-locked signatures of invitation, uncertainty reduction, or movement commitment to the cortical and subcortical systems involved in maintaining broader strategic context. More broadly, the value of this paradigm for neuroscience may lie not in a one-to-one fit with any single recording modality, but in its potential to offer a shared task framework for examining transient neural events, inter-brain coupling, and more stable strategic representations in relation to the same observable behavior.

Importantly, the value of combining the CCF framework with fMRI or EEG-fMRI is not simply that it would permit familiar social variables to be measured with higher spatial or temporal resolution. Rather, it would make it possible to examine a level of neural analysis that is difficult to isolate in conventional round-based neuroimaging paradigms using the same methods: how social decisions are continuously updated during ongoing action as a function of the partner's visible behavior. In such paradigms, neural activity is typically modeled around experimenter-defined events such as offer presentation, choice execution, and feedback. By contrast, in a CCF-based neuroimaging adaptation, critical decisional transitions can emerge from the interaction itself, including the onset of a partner-generated invitation, the uptake or rejection of that invitation, trajectory redirection, convergence on a shared target, and the breakdown or recovery of coordination before final target collection. Although scanner-based implementations would not fully preserve the original setup, particularly its direct face-to-face co-presence, shared displays and live video windows could still preserve aspects of the ongoing partner-dependent information available during interaction. Because such a design would retain more of the interaction-defined structure of social decision-making than conventional round-based paradigms, it could also provide a more ecologically valid basis for neural analysis. In this context, greater ecological validity is important not merely because the task becomes more naturalistic, but because it can improve the validity and interpretability of neural activity analyses by aligning measured brain activity more closely with the social-cognitive processes that the paradigm is intended to capture. Thus, the distinctive contribution of combining CCF with fMRI or EEG-fMRI would be to identify the neural systems and timescales involved in partner-contingent decision updating as interaction unfolds, rather than only in discrete decisions anchored to externally defined trial events.

Across these methodological options, the CCF paradigm provides a rich bridge from behavioral analysis to social neuroscience, combining ecological social interaction with explicit event structure, tractable computational variables, and measurable dyadic coordination. For capturing fast neural dynamics, EEG, MEG, and ECoG are particularly appropriate; for studying interactive social coupling, hyperscanning offers a natural approach; and for network localization and multiscale integration, fMRI and EEG-fMRI remain promising despite practical constraints. By structuring cooperation and competition as ongoing, embodied, and partner-contingent processes, the paradigm establishes a strong foundation for investigating the neural dynamics underlying social decision-making.

## Analyzing fast dyadic neural dynamics in the CCF paradigm

**Figure 1:**
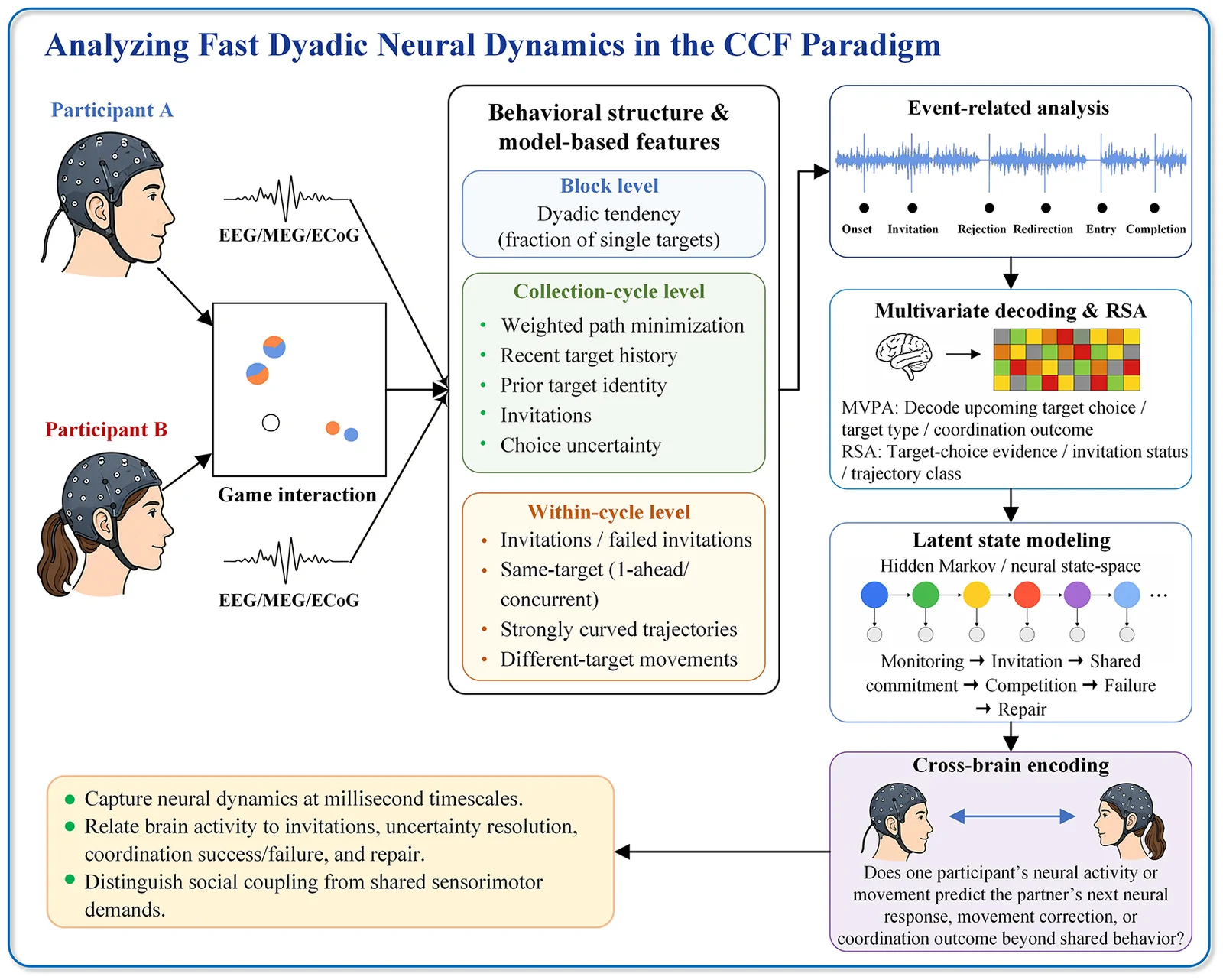
Analytical framework for fast dyadic neural dynamics in the CCF paradigm.

The preceding section outlined how different neural methods could be integrated with the CCF paradigm across multiple temporal and spatial scales. A more focused question, however, is how to analyze the fast neural dynamics that are most directly matched to the paradigm's central behavioral innovation. In the CCF task, socially meaningful changes do not occur only at the level of final target choice or accumulated strategic tendency; they unfold rapidly as participants monitor each other's movement, place or respond to invitations, revise trajectories, and shift between coordinated and uncoordinated states. For this reason, EEG, MEG, and, where clinically feasible, ECoG are especially important extensions of the paradigm. Their main value lies in their temporal precision: they are better suited than slower hemodynamic methods for tracking how neural activity changes during the brief intervals in which uncertainty is reduced, commitment emerges, coordination fails, or corrective action begins. The aim would therefore be not merely to time-lock neural activity to conventional task events such as target onset, target entry, or feedback, but to broaden event-related neural analysis by linking high-temporal-resolution neural data to the richer behavioral and computational variables afforded by the CCF paradigm across multiple timescales. Figure 1 summarizes this proposed analytical framework, linking nested behavioral features in the CCF task to event-related, multivariate, latent-state, and cross-brain neural analyses.

The behavioral structure of the CCF task provides a way to organize neural analysis across nested timescales. Relatively stable dyadic tendencies, indexed by the fraction of single targets, can be treated as a block-level context that modulates neural responses rather than as the primary time-locked regressor. At the collection-cycle level, weighted path minimization and the full GLM could specify how spatial costs, recent target history, prior target identity, sensorimotor invitations, and model-derived choice uncertainty bias the next target choice. At the within-cycle level, spatiotemporal trajectory categories describe how these choice tendencies are expressed in ongoing interaction, including invitations and failed invitations, one-ahead and concurrent movements to the same target, strongly curved trajectories, and movements toward different targets.

This nested organization makes deconvolutional event-related modeling (Pernet et al., 2011) and temporal response function analysis (Lalor et al., 2006) particularly suitable, because both approaches can relate neural activity to temporally overlapping decision and movement processes. Neural responses could be aligned to analytically meaningful transitions, including target appearance, invitation onset, partner uptake or rejection, trajectory redirection, target entry, and collection completion, while continuous kinematic covariates account for movement execution. In this way, neural analysis would move beyond asking whether isolated events evoke activity, and instead test how neural dynamics evolve as a collection cycle shifts from spatially guided target selection to partner-contingent coordination. For example, activity preceding trajectory correction could be examined to determine whether emerging corrections already differentiate invitations that will be accepted, invitations that will fail, and movements that will resolve toward different targets before these outcomes are fully expressed in overt behavior.

The schematic summarizes how neural activity recorded from both participants (e.g., EEG, MEG, or ECoG) can be linked to continuous dyadic behavior across nested timescales in the continuous cooperation-competition foraging (CCF) paradigm. At the block level, relatively stable dyadic tendencies are indexed by the fraction of single-target choices. At the collection-cycle level, behavioral and model-based variables—including weighted path minimization, recent target history, prior target identity, invitations, and choice uncertainty—characterize the evolving decision context. At the within-cycle level, fine-grained trajectory classes such as invitations and failed invitations, same-target approaches, strongly curved trajectories, and different-target movements capture moment-to-moment coordination dynamics. These nested features can be used to organize complementary neural analyses, including event-related modeling, multivariate decoding and representational similarity analysis, latent-state modeling, and cross-brain encoding, to examine how neural activity tracks rapid social events, emerging commitment, coordination success or failure, and partner-contingent prediction beyond shared behavior.

Beyond event-related analysis, the CCF paradigm would also support multivariate and state-based neural modeling. Time-resolved decoding could test whether EEG/MEG activity, and where available ECoG activity, predicts the upcoming target choice, target type, or coordination outcome before it becomes evident in the cursor trajectory. Representational similarity analysis could examine whether neural pattern geometry is organized primarily by strategic regime, target-choice evidence, invitation status, or trajectory class. In parallel, hidden Markov (Kemere et al., 2008) or neural state-space models (Zoltowski et al., 2020) could be used to characterize transitions among latent interaction states, such as monitoring, invitation, shared commitment, competition, coordination failure, and repair. This would be especially informative because the behavioral findings suggest that uncertainty does not necessarily lead to breakdown; in some cases, dyads preserve coordination through signaling and online trajectory adjustment.

Finally, cross-brain encoding models could examine whether signals from one participant help predict what the other participant does next. More specifically, these models could test whether one person's neural activity or movement trajectory predicts the partner's subsequent neural response or movement correction. The key question is whether this cross-partner information improves prediction of meaningful coordination outcomes after accounting for what is already visible in behavior, such as each participant's target choice, recent interaction history, and the timing and similarity of their movements. These outcomes include whether an invitation is accepted, whether uncertainty about the next action is resolved, whether an emerging coordination failure is repaired, and whether both participants ultimately converge on the same target. Yet this analysis also exposes a critical interpretive problem: in a task where partners often move at similar times and along related trajectories, apparent inter-brain coupling may partly arise from shared sensorimotor demands rather than genuine social interaction. This concern motivates a broader methodological issue for future neural implementations of the CCF paradigm: how to distinguish social coupling from motor entrainment.

## Disentangling social coupling from motor entrainment

A central methodological issue in the CCF paradigm concerns the need to distinguish social coupling from shared sensorimotor dynamics. In the task, participants often share aspects of movement timing and kinematic structure, and these shared sensorimotor dynamics can shape neural signals independently of social prediction or reciprocal adaptation. This concern is reinforced by behavioral findings showing that competitive dyads move faster and follow distinct trajectory profiles (Lewen et al., 2025), indicating that condition-related differences in movement may complicate the interpretation of neural effects across measures, not only in inter-brain synchrony analyses.

For this reason, neural effects should be evaluated while accounting for key motor variables, including movement velocity, trajectory curvature, and reaction-time asymmetry. Such variables can be derived from available behavioral measurements, including high-temporal-resolution task records and, where available, video-based motion tracking or pose estimation, providing an additional index of ongoing bodily dynamics during interaction. Accounting for these factors is necessary to test whether the observed neural patterns reflect social coupling *per se*, rather than common kinematic structure or parallel responses to the same task demands. This point is especially important in hyperscanning, where shared movement can directly inflate estimates of inter-brain coherence, but the same logic extends to more general analyses of task-evoked or time-resolved neural activity.

However, in the interactive task (Lewen et al., 2025), movement is not merely an external nuisance variable superimposed on a social process; it is one of the primary channels through which social information is generated and exchanged. Participants communicate commitment, hesitation, adaptation, and competition through the timing and geometry of their actions. Motor and social processes are therefore often intrinsically intertwined, making it difficult to determine whether shared neural structure reflects common kinematics, social coordination, or both. This problem is further compounded by the fact that motor variables in naturalistic interaction are high-dimensional, temporally evolving, and often nonlinearly related to neural activity. Because these variables are frequently correlated with the social conditions of interest, statistical attempts to separate them can be unstable and difficult to interpret. More sophisticated approaches, including the extraction of kinematic information from video recordings, may improve the measurement of bodily dynamics, but they do not resolve the underlying problem. Accordingly, incorporating motor variables can strengthen the interpretation of shared neural structure, but it cannot fully disentangle social coordination from the movements through which it is enacted.

## Reproducibility and statistical robustness in continuous naturalistic paradigms

One methodological challenge raised by continuous and naturalistic paradigms concerns reproducibility. Compared with conventional trial-based games, the CCF paradigm generates temporally extended, high-dimensional, and interdependent behavioral data. This richness is precisely what makes the paradigm valuable, but it also increases the number of analytic decisions, including how to define interaction events, segment trajectories, model temporal history, control for motor variables, and evaluate dyad-level variability. Consequently, reproducibility in such paradigms depends not only on whether the apparatus and payoff structure can be recreated, but also on whether independent analyses make comparable choices when transforming continuous movement streams into discrete behavioral events and model predictors. In the CCF paradigm, this concern is especially relevant to the intermediate analytic steps through which movement trajectories are made interpretable, such as specifying the distance-based choice model, deciding how recent choices and sensorimotor cues enter the analysis, and assigning trajectories to categories such as invitations, failed invitations, concurrent movements, strongly curved paths, or choices of different targets. These decisions would be unlikely to undermine the general value of the paradigm, but they could affect more specific claims about whether a given behavioral pattern primarily reflects cooperation, competition, recent-history dependence, uncertainty reduction, or online sensorimotor adjustment.

Closely related to this is the issue of statistical robustness. In continuous dyadic tasks, observations recorded close together in time are temporally dependent, while the behavior of the two members of a dyad is mutually coupled. Treating these samples as independent may therefore produce overly precise estimates and make conclusions more sensitive to analytic decisions than the nominal sample size suggests. Future studies could reduce this risk by preregistering primary outcomes, exclusion criteria, and model-comparison criteria, and by reporting sensitivity analyses that test whether the main findings remain stable across reasonable variations in convergence windows, final-segment thresholds, and trajectory-classification rules. Dynamic hierarchical models, which estimate temporal processes within dyads while allowing these processes to differ systematically across dyads, would provide a suitable framework for this purpose (Asparouhov et al., 2018; Hamaker et al., 2018). State-space models could further examine whether coordination changes gradually over time or shifts more abruptly between latent interaction states (Durbin and Koopman, 2012). These approaches may provide a useful methodological direction for future research on how uncertainty-related coordination unfolds over time.

## Extending the paradigm across social and clinical contexts

Beyond laboratory dyads, the CCF paradigm offers a flexible platform for examining how social relationships modulate continuous cooperation-competition dynamics. For example, comparing romantic partners, strangers, or hierarchical dyads could reveal how relational priors shape strategy stabilization and inter-brain coupling regimes.

Similarly, extending this framework to clinical populations (e.g., autism spectrum conditions or social anxiety) may reveal whether atypical social functioning reflects altered stability of interpersonal attractor states, reduced invitation sensitivity, or disrupted neural coupling transitions.

## Conclusion

Lewen et al. (2025) do more than introduce an elegant task; they reposition cooperation and competition as phenomena that must be studied within continuous, embodied social interaction. By embedding decision-making in continuous time, shared space, mutual visibility, and ongoing perception-action coupling, the CCF paradigm moves beyond the static logic of trial-based economic games and captures social behavior as an evolving interpersonal process rather than a sequence of isolated choices.

The significance of this contribution is two-fold. At the behavioral level, the CCF paradigm makes it possible to observe how cooperative and competitive tendencies emerge, stabilize, break down, and reorganize over time through continuous movement, reciprocal adjustment, and path-dependent interaction history. At the neuroscientific level, it offers an unusually rich platform for linking neural activity to socially meaningful microdynamics, including invitation signals, uncertainty reduction, coordination breakdowns, and the gradual formation of shared strategic structure. More broadly, the paradigm creates an important bridge between ecologically grounded social behavior and the growing effort to study its neural implementation with event-related, continuous, and hyperscanning approaches.

For these reasons, the CCF paradigm should be regarded as an important step toward an interaction-level science of social decision-making. Rather than treating cooperation and competition as fixed properties of isolated individuals, it encourages a shift toward understanding them as dynamically organized states emerging between interacting agents. The broader promise of this work lies not simply in improving the measurement of dyadic outcomes, but in helping explain how shared behavioral states are continuously formed, maintained, and transformed in real time.
